# Supplementary material for: The causal relationship between the human gut microbiota and pyogenic arthritis: a Mendelian randomization study
Source: Front Cell Infect Microbiol. 2024 Nov 26;14:1452480. doi: 10.3389/fcimb.2024.1452480 (PMC11629706; doi:10.3389/fcimb.2024.1452480)
Supplement: Supplementary file 1 [file DataSheet1.zip › mendelian test/ebi-a-GCST90027808.csv_forest.pdf]

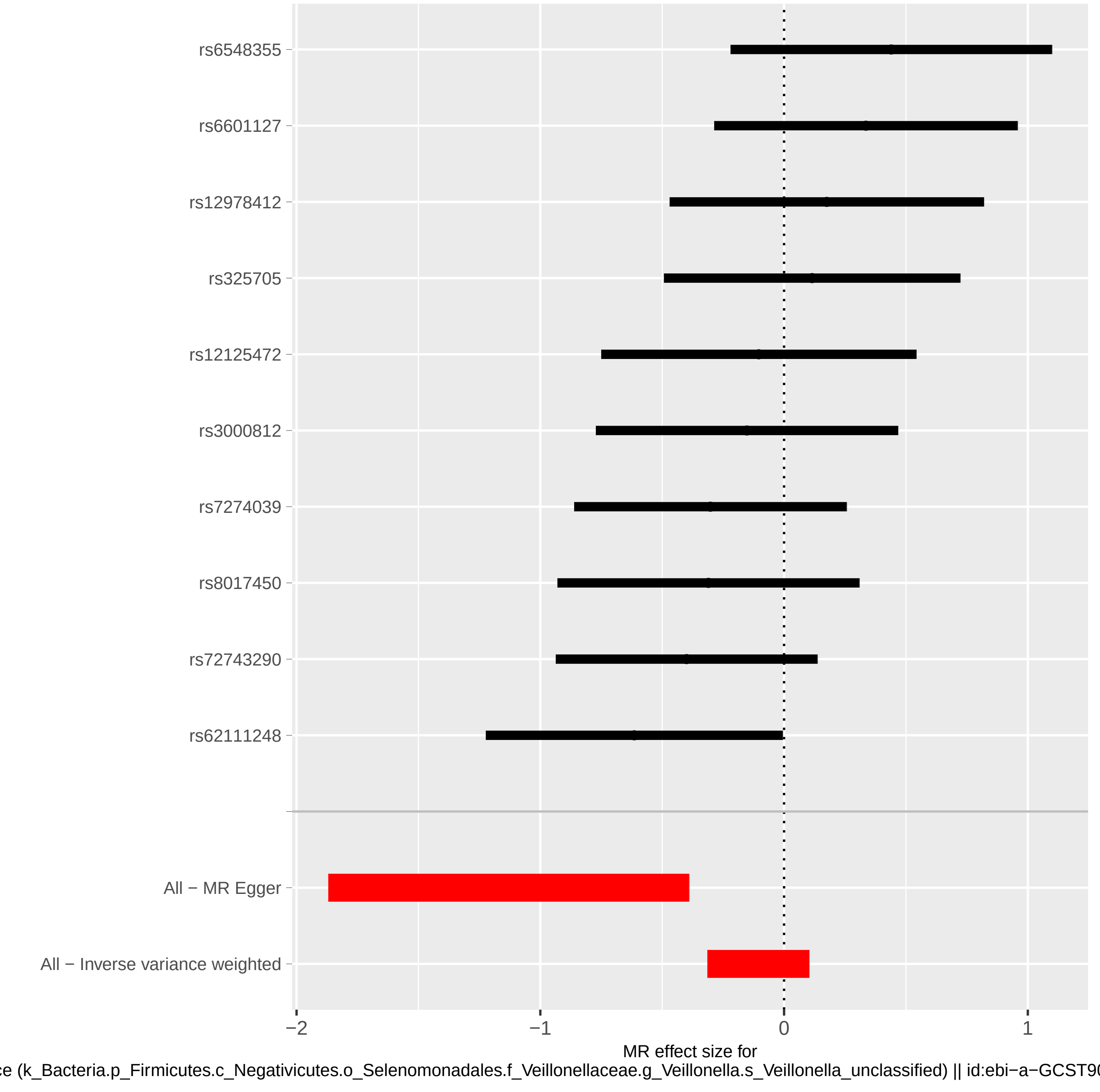
rs6548355
rs6601127
rs12978412
rs325705
rs12125472
rs3000812
rs7274039
rs8017450
rs72743290
rs62111248
All − MR Egger
All − Inverse variance weighted
−2
−1
0
1
MR effect size for
ce (k_Bacteria.p_Firmicutes.c_Negativicutes.o_Selenomonadales.f_Veillonellaceae.g_Veillonella.s_Veillonella_unclassified) || id:ebi−a−GCST90
